# Air-Coupled and Resonant Pulse-Echo Ultrasonic Technique

**DOI:** 10.3390/s19102221

**Published:** 2019-05-14

**Authors:** Tomás Gómez Álvarez-Arenas, Jorge Camacho

**Affiliations:** Instituto de Tecnologías Físicas y de la Información (ITEFI), Spanish National Research Council (CSIC), 28006 Madrid, Spain; j.camacho@csic.es

**Keywords:** air-coupled ultrasound, ultrasonic NDT, air-coupled transducers, air-coupled pulse-echo, pipe NDT, pipe wall gauge

## Abstract

An ultrasonic, resonant, pulse-echo, and air-coupled nondestructive testing (NDT) technique is presented. It is intended for components, with regular geometries where it is possible to excite resonant modes, made of materials that have a high acoustic impedance (*Z*) and low attenuation coefficient (*α*). Under these conditions, these resonances will present a very large quality factor (*Q*) and decay time (*τ*). This feature is used to avoid the dead zone, produced by the echo coming from the first wall, by receiving the resonant echo from the whole specimen over a longer period of time. This echo is analyzed in the frequency domain to determine specimen resonant frequency, which can be further used to determine either velocity or thickness. Using wideband air-coupled transducers, we tested the technique on plates (steel, aluminum, and silicone rubber) by exciting the mode of the first thickness. As expected, the higher the *Z* and the lower the *α*, the better the technique performed. Sensitivity to deviations of the angle of incidence away from normal (±2°) and the possibility to generate shear waves were also studied. Then, it was tested on steel cylindrical pipes that had different wall thicknesses and diameters. Finally, the use of this technique to generate C-Scan images of steel plates with different thicknesses was demonstrated.

## 1. Introduction

Air-coupled ultrasound is a convenient method for material characterization and nondestructive testing (NDT) when conventional techniques based on water immersion, local immersion, gel coupling, or dry coupling cannot be used. There can be different reasons for the need to use air-coupled techniques. In the case of material characterization, examples are commonly found in the determination of elastic and viscoelastic constants. Examples are also found in determining the microstructural properties of porous, open-pore materials or soluble materials, where the use of coupling fluids must be avoided. In other cases, coupling fluids cannot be used because they can potentially contaminate or modify the material. Examples commonly appear in the food industry and in the study of biological tissues, synthetic membranes, and porous solids. Similarly, air-coupled ultrasound can be a very interesting solution for NDT (see [1] for an early review in this field). Examples correspond to cases where the potential penetration of fluids within the testing piece is to be avoided. In other cases, air-coupled techniques can be used to replace water-jet or water immersion systems when saving water, time, and/or energy consumption is advantageous. Finally, air-coupled techniques can be thoroughly used to generate and receive guided waves in solid structures. This may present some advantages in order to test large specimens or those that present difficult access points. Use of air-coupled ultrasound for Lamb waves in metal plates was first demonstrated in 1973 [2]. Since then, Lamb waves have been extensively used for different materials, including fiber-reinforced polymer composites [3].

The main problem of air-coupled ultrasound is the huge impedance mismatch between air and any solid material. This impedance mismatch gives rise to a very high reflection coefficient (*R*) at any air/solid interface (*R* ≈ 1). Thus, only a very small portion of incident energy is transmitted (*T* ≈ 0, where *T* is the transmission coefficient), which leads to very low-amplitude transmitted signals and poor signal-to-noise ratio (*SNR*) values. This same problem also affects piezoelectric air-coupled transducers, which make the situation even more difficult. In most cases, there is also a significant difference between the ultrasonic velocity in the air and in the solid material, which produces a strong refraction at the interfaces; consequently, the limit angle will appear at quite low values.

Despite all these problems, and because of the continuous improvement in air-coupled transducers [4], electronics, signal-processing-like coded excitation [5,6], and inspection techniques, there are good records of successful applications in different fields and by different groups [7].

One of the consequences of having *R* ≈ 1 is that energy will be strongly confined within both air cavities and solid pieces. Depending on the air cavity, solid piece, and ultrasonic field geometry this can lead to the appearance of strong resonances. Thickness resonances in plates at normal incidence can be used as a way to boost the level of transmitted energy through the plate, especially for high-impedance materials [8]. Later, use of these resonances was proposed by Hutchins and coworkers as a means to determine velocity in the material if thickness was known [9,10] and, alternatively, to determine the thickness of metal plates [11]. Later advances in this sense included the use of oblique incidence to obtain information about shear properties [12,13], the use of both magnitude and phase spectra to obtain simultaneous information on thickness and velocity [14], and the analysis of layered composites using resonance imaging [15]. 

Given that *R* ≈ 1, air-coupled ultrasound can be well-used in pulse-echo mode to study the roughness of surfaces, to produce an image of the surface profile [16,17], and as a scanner for Braille [18]. One of the main limitations of air-coupled ultrasound in studying the inner volume of a solid is that it is impossible, in most practical situations, to operate in pulse-echo mode, although this possibility has been considered since the early developments of this technique [19]. This is because the echo coming from the back surface is very small and appears buried within the echo reflected from the front surface. The only chance to detect this low-amplitude, back wall echo is to avoid the dead zone produced by the front wall echo. This implies that we must wait until its influence disappears, a time we call *t_DZ_*. Considering that, in most air-coupled applications, low frequencies (<0.5 MHz) and relatively narrowband transducers are commonly used, *t_DZ_* can be quite large; this limits the applicability of the technique to quite thick plates. Unfortunately, this imposes another restriction, as the attenuation coefficient in the material must be low enough. In addition, distance between the transducer and the front plate surface must be large enough so that the reverberations within the air column do not overlap with the back wall echo. This normally implies a long propagation path in the air, which introduces some extra losses from attenuation in the air and beam diffraction. Exceptions appear when *t_DZ_* is relatively shorter; this is the case in low-impedance solids (porous or soft solids) or when the pressure in the air is elevated (pressurized pipes) [20,21].

An alternative can be the use of two different transducers in pitch–catch mode, but the dead zone does not disappear completely, and the technique becomes more difficult to implement since two different transducers are involved [22]. If oblique incidence is considered, accuracy in the control of angle and location is critical in order to separate specular reflection from the front surface and back wall echoes originating from longitudinal and shear waves, which also requires accurate knowledge of the longitudinal and shear wave velocities in the material.

For plates made of high-impedance (*Z*) and low-attenuation coefficient (*α*) materials (like most metals, glass, and some ceramics) the *Q-factor* of the thickness resonance can be very large, which means that the time the signal keeps reverberating within the material can be very large as well. The proposed idea in this paper is to launch a wideband, airborne, ultrasonic pulse towards a solid material (with high *Z* and low *α* and at normal incidence) in order to excite a high-*Q* resonance of the solid piece. Then, the same transducer is used to receive the resonant echo generated by the solid. Because of the high *Q-factor* of the resonance in the test object, this can be done after a long enough time so that the front wall echo and its reverberations within the air gap have disappeared. Clearly, the transducer frequency band must encompass at least one of the resonant frequencies of the solid specimen so that the pulse is able to generate resonances in it. 

The objective of this paper is twofold: (i) to test the feasibility of this approach in plates (normal incidence) and cylindrical pipes (quasi-normal incidence) made of different materials, and (ii) to test the possibilities of this technique to generate a C-Scan image, based on the value of the resonant frequency of the plate/pipe, so that the thinning of plate or pipe walls can be determined for NDT applications.

## 2. Materials and Methods

### 2.1. Methods

The proposed methodology consisted of selecting a set of plates and pipes made of different materials and with different thicknesses. First, they were measured using a conventional air-coupled through-transmission (TT) configuration using normal incidence, wideband transducers, and spectral analysis of the transmission coefficient with the purpose to determine the frequency location of the first thickness resonance (*f_res_*). For plates at normal incidence, *f_res_* is derived directly from the plate thickness (*t*) and the phase velocity of ultrasound longitudinal waves in the material (*v*) by:

(1) $$f_{res}=\frac{v}{2t}.$$

Other parameters of interest for this study included the duration of the reverberations within the material, as this provided an initial estimation of the feasibility of the proposed resonant pulse-echo procedure. This was related with the quality factor of the resonance (*Q*-factor or *Q*), which was determined by *Z* of the solid specimen and the air, *α* of the solid, and other factors like the plate surface roughness, the presence of plate imperfections (lack of homogeneity of the solid or lack of parallelism between both plate surfaces), and any deviation of the beam away from normal incidence. It is defined by:

(2) $$Q=\frac{f_{res}}{{\Delta f|}_{6dB}},$$

where ${\Delta f|}_{6dB}$ is the width of the resonance peak at 6 dB below the maximum value. The *Q* factor is related to the decay time (*τ*) by:

(3) $$\tau =\frac{Q}{\pi f_{res}}.$$

After the measurement in TT mode, we switched to the pulse-echo (PE) configuration, and we took the Fast Fourier Transform (FFT) of the signal for a temporal window located at *t*_0_ > *t_DZ_* to verify if there was still some measurable contribution of the reverberations in the plate from the thickness resonances. This was done by comparing the frequency components of the FFT with that measured in the TT configuration. In some cases, we took different windows at different *t*_0_ values to check the integrity of the results. This process was repeated for samples having different thicknesses, made of different materials, and with different values of the *Q* factor, *τ*, and *f_res_*. 

Then, the influence of the angle of incidence was tested using steel plates with the purpose to estimate how critical the transducer–plate alignment was and if it was possible to generate and observe shear waves. This was done by measuring variations in the amplitude of the thickness resonance peak, measured in pulse-echo mode, when the angle of incidence varied from −2 to 2 degrees.

Once the feasibility, the robustness, and the limits of the technique were tested with plates, we followed the same procedure with two different cylindrical pipes made of two different steels and with different thicknesses. 

Finally, we performed a C-Scan image using the proposed PE-resonant technique of steel plates with different thicknesses and used the value of the resonant frequency to generate a C-Scan image.

### 2.2. Experimental Methods and Setups

**Figure 1:**
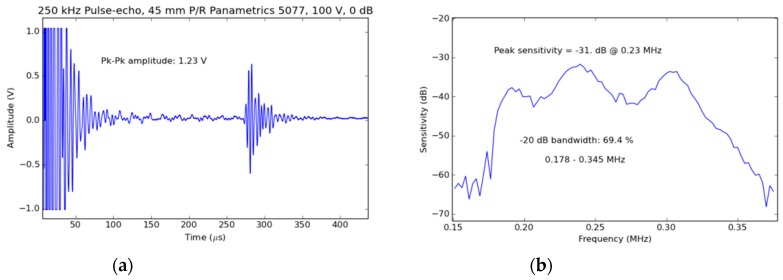
Transducer impulse response in the time domain V_RX_ (**a**) and sensitivity frequency band SNS (**b**) in pulse-echo (PE) mode. The FFT was obtained from V_RX_ in a temporal window between 250 and 450 μs.

All the results presented in this work were obtained using wideband, high-sensitivity air-coupled transducers with a center frequency of 0.25 MHz, a −20 dB bandwidth (60%), a circular aperture (diameter = 25 mm), and a flat radiating surface that was designed and built in our research group [23]. These transducers could be used both in TT or PE configurations. Signals in the time domain and sensitivity (*SNS*) in the PE mode are shown in Figure 1. For the reflector we used a brass block, and distance to reflector was 45 mm. *SNS* is obtained by:

(4) $$SNS\left(dB\right)=20log_{10}\frac{\left|\mathrm{FFT}\left(V_{Rx}\right)\right|}{\left|\mathrm{FFT}\left(V_{Tx}\right)\right|},$$

where *V* is the electrical voltage measured at transducers terminals, and FFT denotes the fast Fourier transform. The temporal window for the FFT was square, with a duration (Δ*t*) 200 µs, and was located at *t*_0_ = 0 µs and *t*_0_ = 200 µs for $\mathrm{FFT}\left(V_{Tx}\right)$ and $\mathrm{FFT}\left(V_{Rx}\right)$, respectively. 

The response shown in Figure 1 was obtained by driving *Tx* (*V_TX_*) with a negative semicycle of square pulses, amplitude of 100 V, and a frequency of 250 kHz, provided by an Olympus 5077PR pulser-receiver (Tokyo, Japan). Signals generated at *Rx* (*V_RX_*) were sent to the receiver stage of the 5077PR. The gain was set to 0 dB with no filtering, and the signal was transferred to a digital oscilloscope (Tektronix 5072, Beaverton, OR, USA) to digitize the signal, display it, store it, and extract it to the FFT.

#### 2.2.1. Through-Transmission (TT)

**Figure 2:**
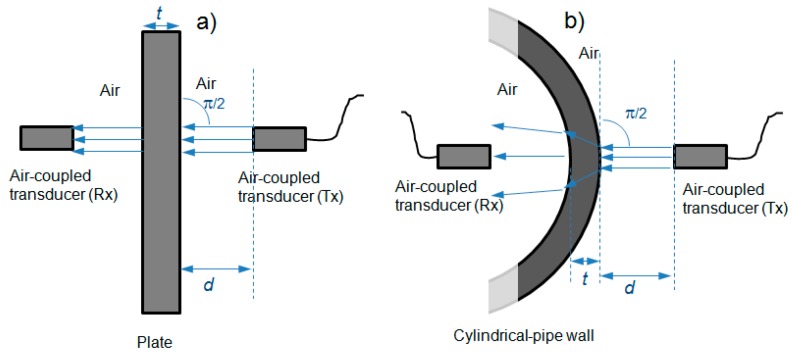
Transducers and sample configurations in through transmission measurements: (**a**) plate and (**b**) cylindrical pipe.

Transducers and sample configurations for both plates and pipes are shown in Figure 2. An Olympus 5077 (Tokyo, Japan) pulser-receiver was used to drive *Tx* with a square semicycle (pulse amplitude: 400 V, pulse center frequency: 250 kHz, and pulse repetition frequency (PRF): 100 Hz) and to amplify and filter the signal provided by *Rx* (gain: +59 dB, low-pass filter: 10 MHz). It was then transferred to the digital scope (Tektronix 5072, Beaverton, USA, bandwidth limit set to 20 MHz), which was triggered by the synchronism signal provided by the 5077PR. The scope digitized the signal and then was loaded into MATLAB (Mathworks, Natick, MA, USA) for further processing. *Tx – Rx* separation was typically 120 mm. Normal incidence was used for plates, while for cylindrical pipes, transducers were aligned along the radial direction (see Figure 2). 

#### 2.2.2. Pulse-Echo (PE)

**Figure 3:**
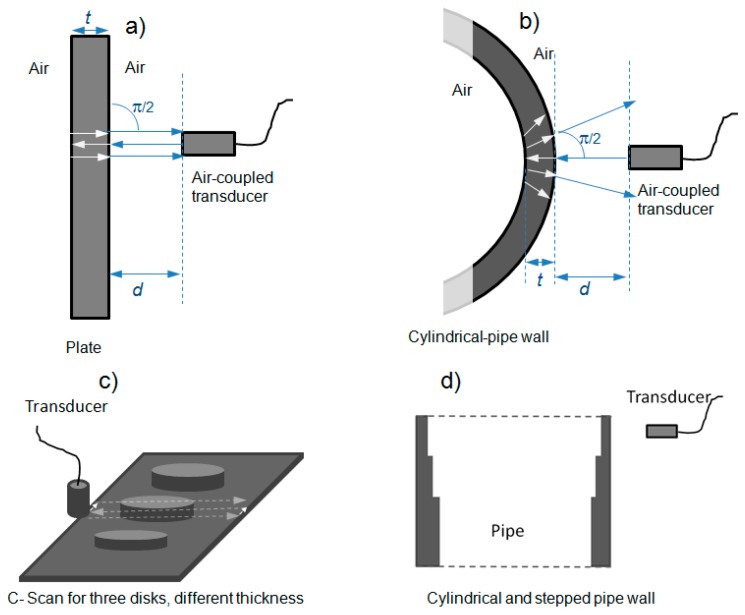
Transducers and sample configurations in pulse measurements: (**a**) plate, (**b**) cylindrical pipe, (**c**) C-Scan of three different steel disks, and (**d**) cylindrical pipe with stepped pipe wall thickness.

Transducers and sample configurations are shown in Figure 3. The same configuration as before was employed, with the exceptions that now we operated in PE mode and that a lower pulse repetition frequency (PRF) was used (25 Hz). The trigger signal was provided by an Agilent 3320A (Santa Clara, CA, USA) arbitrary waveform generator; this trigger signal was employed to trigger both the 5077 pulser-receiver and the oscilloscope.

#### 2.2.3. Resonant PE C-Scan Images

The configuration of Figure 3c was used to scan three stainless steel disks of different thicknesses (10.0 mm, 11.0 mm, and 12.0 mm) placed on a planar surface. A DIS-300 automated scanner and an Air-Scope ultrasound system, both from Dasel S.L. (Madrid, Spain), were used to acquire reflection signals from a single transducer located at a distance of 40 mm. A grid of 50 mm × 125 mm was scanned in 1 mm steps (X and Y directions). The encoders of the scanning system were used to trigger pulse-echo acquisitions of 7 ms windows, which were delayed 4 ms from the excitation signal. Acquired data were loaded in Matlab, and the center frequency of each signal was estimated by FFT in a temporal window at *t*_0_ > *t_DZ_.* Finally, a C-Scan was generated by color coding the resonant frequency in Hz.

### 2.3. Materials

Descriptions of the main properties of the samples employed for the study are shown in Table 1 and Table 2. The diameters of the steel pipes were selected to be much larger than the transducer’s diameter (25 mm) so that we were close to quasi-normal incidence. The wall of the 305 mm diameter steel pipe was initially uniform and equal to 12.59 ± 0.4 mm; it was reduced along three 40 mm-wide bands using a lathe (see Figure 3d and Table 2) to generate four sections with different thicknesses. In these cases, the lathed surfaces presented a larger roughness that certainly affected the amplitude of the resonant mode of the pipe wall in these sections. Finally, three steel discs of 36 mm diameter (same steel as in Table 1) and different heights (10.0 mm, 11.0 mm, and 12.0 mm) were used for the C-Scan (see Figure 3c).

## 3. Results

### 3.1. Through-Transmission

**Figure 4:**
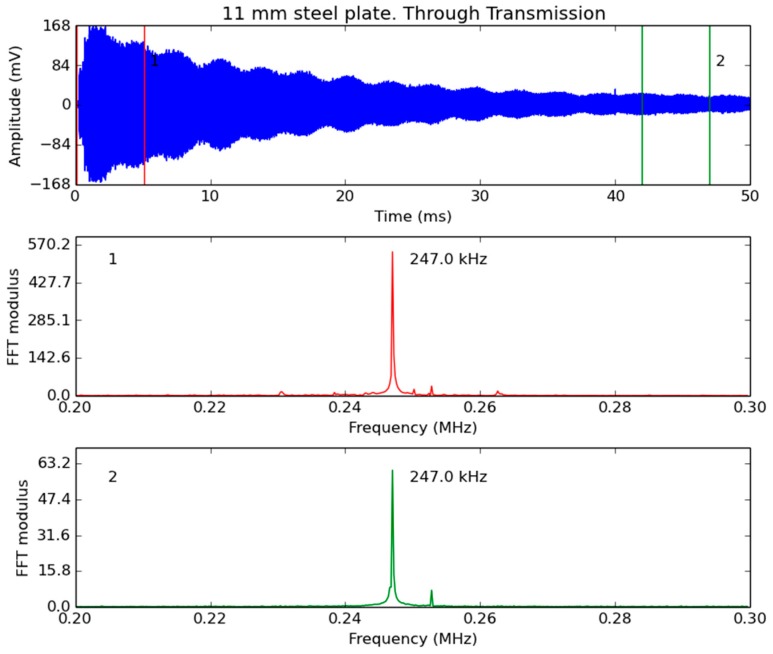
Through-transmission in the 11 mm-thick stainless steel plate. Top: signal in the time domain. Center: FFT modulus of the signal within window (1). Bottom: FFT modulus of the signal within window (2).

For the steel plates in Table 1, and because of the very large *Z* mismatch between the air (~420 Rayl) and the steel as well as the expected low *α* in the steel plate at the resonant frequency, we expected *R* ≈ 1 and very large values for both *Q*-factor and *τ*. As an example, transmitted signal and FFT measured in the 11 mm steel plate are shown in Figure 4. The transmitted signal in the time domain was much larger than the incident one (Figure 1). This was because the reverberations within the plate were almost free of damping and gave rise to a strong and sharp thickness resonance. FFT of the signal revealed that $f_{res}$ = 247.06 kHz, *Q* > 6200, and *τ* > 8 ms. Then, according to Equation (1), *v* = 5435 m/s; considering *ρ* = 7800 kg/m^3^, then *Z* = 42.4 MRayl, and *T* = 3.72 × 10^−5^. Actually, the decay time of the reverberations in the plate was so long that it was possible to place a 5 ms temporal rectangular window, at a time called *t*_0_, where *t*_0_ = 42 ms, and get the same frequency content of the signal when a similar window was located at *t*_0_ = 0.02 ms (see Figure 4) with a 19 dB loss in the peak amplitude of the FFT. Similar results were obtained for the other stainless steel plates. Table 3 shows the measured thickness resonant frequencies for all cases. From Equation (1) we knew that the ratio of the resonant frequencies (*f*_1_ and *f*_2_) of two plates of the same material, but with different thicknesses (*t*_1_ and *t*_2_), was: *f*_1_/*f*_2_ = *t*_2_/*t*_1_. In this case, this relationship was upheld, as we had *t*_2_/*t*_1_ = 1.09, 1.1, and 1.2, and the measured ratios of the resonant frequencies were *f*_1_/*f*_2_ = 1.088, 1.101, and 1.198, respectively.

**Figure 5:**
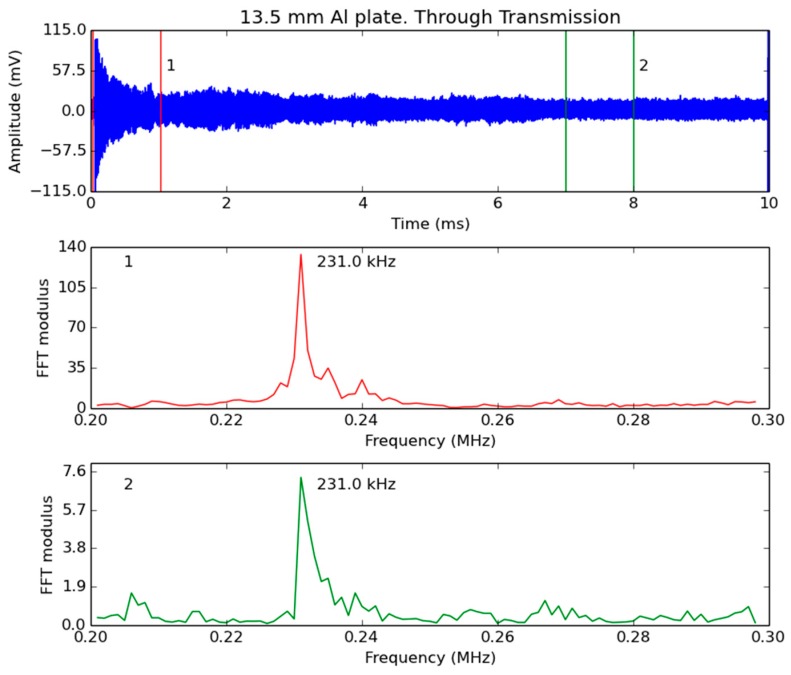
Through-transmission in the 13.5 mm-thick aluminum plate. Top: signal in the time domain. Center: FFT modulus of the signal within window (1). Bottom: FFT modulus of the signal within window (2).

Compared with stainless steel plates, a lower *Q* value for the first thickness resonance was expected in aluminum plates because of the lower value of *Z* and a larger *α*. Results for a 13.5 mm-thick plate are shown in Figure 5. $f_{res}$ = 231 kHz, *Q* > 900, and *τ* > 1.2 ms. According to Equation (1) and considering *ρ* = 2700 kg/m^3^, then *v* = 6237 m/s, and *Z* = 16.7 MRayl; hence, *T* = 9.8 × 10^−5^. That is, a much more attenuated resonance (compared with the previous case) was obtained. In spite of lower *Q* and *τ* values, it was still possible to take a delayed (*t*_0_ = 7 ms) 1 ms-long temporal window and obtain the same frequency content of the signal as when a nondelayed window was used. The amplitude reduction in the FFT peak produced by delaying the window was now larger than in the case of steel plates (even considering that now *t*_0_ was also much smaller) at 25 dB.

**Figure 6:**
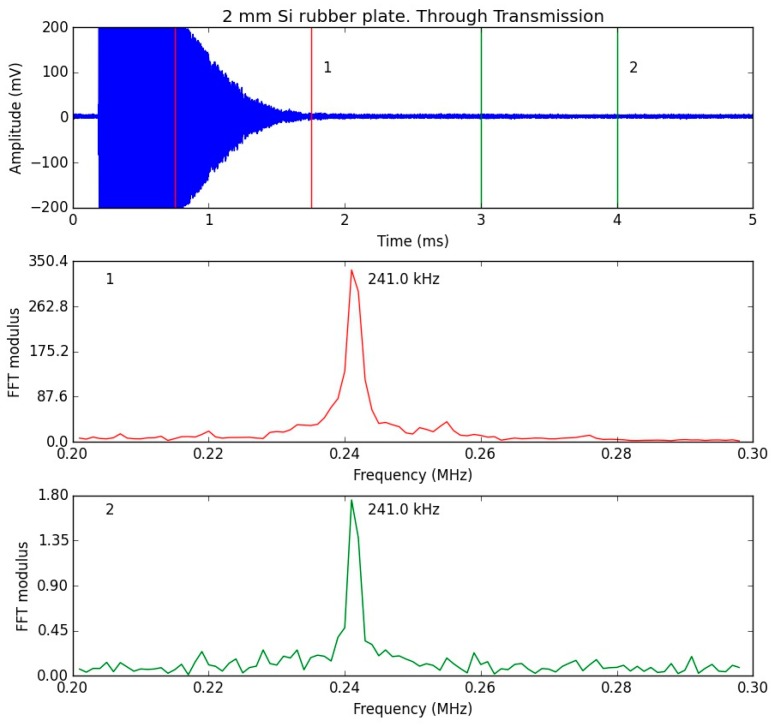
Through-transmission in the 2 mm-thick rubber plate. Top: signal in the time domain. Center: FFT modulus of the signal within window (1). Bottom: FFT modulus of the signal within window (2).

For a silicone rubber plate, and because of the even lower *Z* and larger *α* of this material, the *Q* factor and the decay time of the thickness resonance were expected to be much lower. These features are shown in Figure 6: $f_{res}$ = 241.7 kHz, *Q* ≈ 198, and *τ* ≈ 0.26 ms. Hence, *v* = 967 m/s, and considering *ρ* = 900 kg/m^3^, then *Z* = 0.87 MRayl, *R* = 0.9981, and *T* = 1.89 × 10^−3^. However, even in this case it was possible to place a 1 ms window (delayed 3 ms) and get the same frequency components of the signal, although the signal amplitude decay (46 dB) was much larger than in the previous cases.

**Figure 7:**
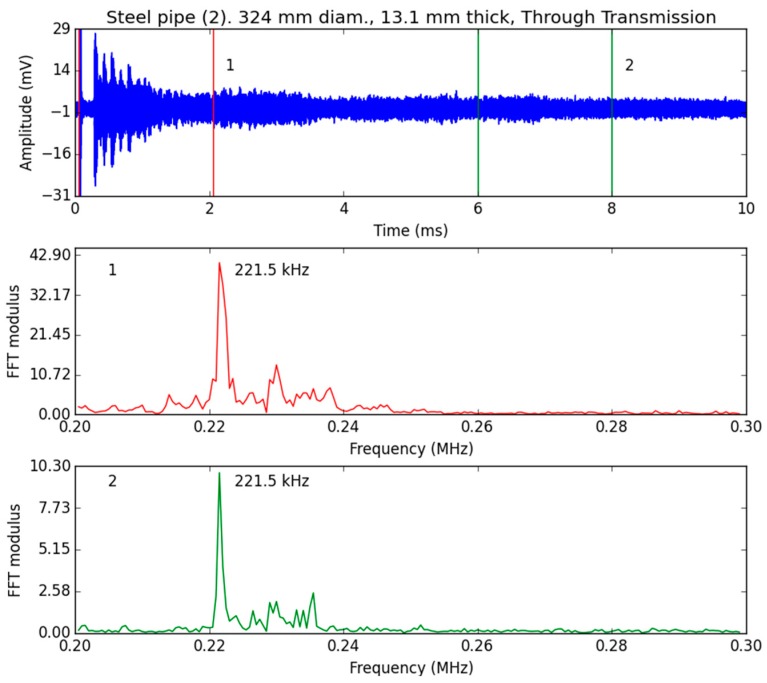
Through-transmission for the 324 mm diameter and 13.1 mm-thick steel pipe. Top: signal in the time domain. Center: FFT modulus of the signal within window (1). Bottom: FFT modulus of the signal within window (2).

Finally, for a steel pipe, and in spite of the high *Z* and low *α* of the material, a much more damped resonance than in the plate specimens was expected. This was because a mode conversion (longitudinal to shear) produced by the non-normal incidence was present. And as the acoustic field reverberated within the pipe wall, the propagation direction was modified, and a reduced portion of the acoustic beam went back into the transducer. One example is shown in Figure 7, where *Q* = 228, while for a steel plate *Q* > 6200. In spite of this larger damping, it was still possible to place a 1 ms window at *t*_0_ = 3.5 ms and obtain the same signal frequency content as for a window located at *t*_0_ = 0.1 ms; the loss in the peak amplitude of the FFT was much lower than in the case of the silicone rubber plate at 13 dB.

Results (materials, sample geometry, thickness, and resonant frequency) for through-transmission are summarized in Table 3.

### 3.2. Pulse-Echo

#### 3.2.1. Plates

**Figure 8:**
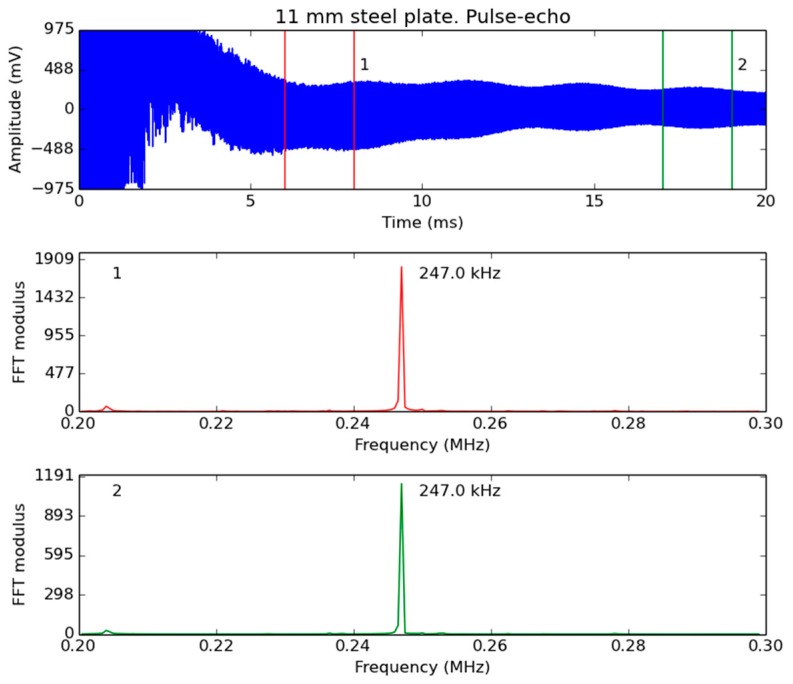
Pulse-echo; 11 mm-thick stainless steel plate. Top: signal in the time domain. Center: FFT modulus of the signal within window (1). Bottom: FFT modulus of the signal within window (2).

As an example of the received signal in PE configuration, Figure 8 shows the result for the 11 mm steel plate (same plate as in Figure 4) using a PRF = 50 Hz (a weakly damped monochromatic signal produced by the high-*Q* thickness resonance in the steel plate). *t_DZ_* expanded up to *t_DZ_* ≈ 5 ms. As amplitude scale was set to measure the signal after *t_DZ_*, the echo from the front face and the reverberations in the air cavity between transducers appeared saturated. Placing a 2 ms temporal window at *t*_0_ > *t_DZ_* and extracting the FFT, the obtained resonant frequency was the same as that obtained using the TT configuration (Figure 4). Moreover, it was possible to delay this temporal window up to *t*_0_ = 17 ms and obtain the same frequency, with a reduced loss in the peak amplitude of only 4 dB (Figure 8).

**Figure 9:**
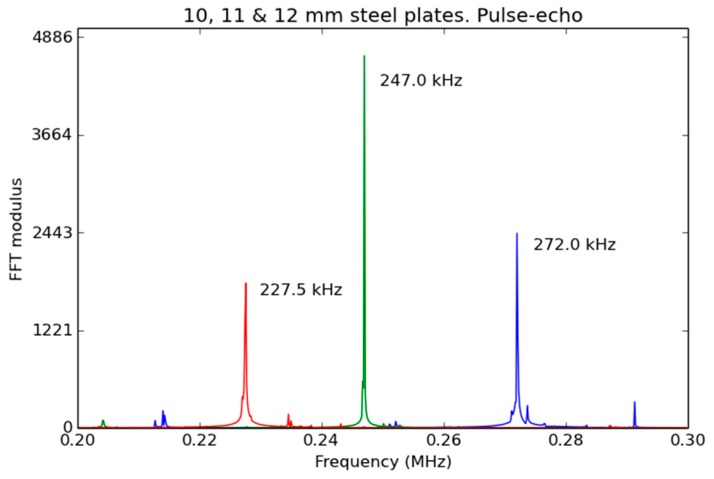
FFT modulus of the signal received from the three stainless steel plates in pulse-echo. Red ($f_{res}$ = 227.5 kHz): 12 mm-thick plate, Green *(*$f_{res}$ = 247.0 kHz): 11 mm-thick plate, and Blue ($f_{res}$ = 272.0 kHz): 10 mm-thick plate. FFT window: *t*_0_ = 10 ms, Δ*t* = 7 ms.

Using this same procedure, we measured resonant frequency and amplitude of the FFT peak for the rest of the samples in Table 1 and Table 2. Figure 9 compares the FFT of the signal received in PE configuration from the three stainless steel plates (10, 11, and 12 mm) using a square temporal window for the FFT located at *t*_0_ = 10 ms and a length Δ*t* = 7 ms. Table 4 shows all measurements using a square temporal window for the FFT of the same length or duration, given by Δ*t*, where: Δ*t* = 2 ms, is located just after *t_DZ_* (which varied for different samples).

**Figure 10:**
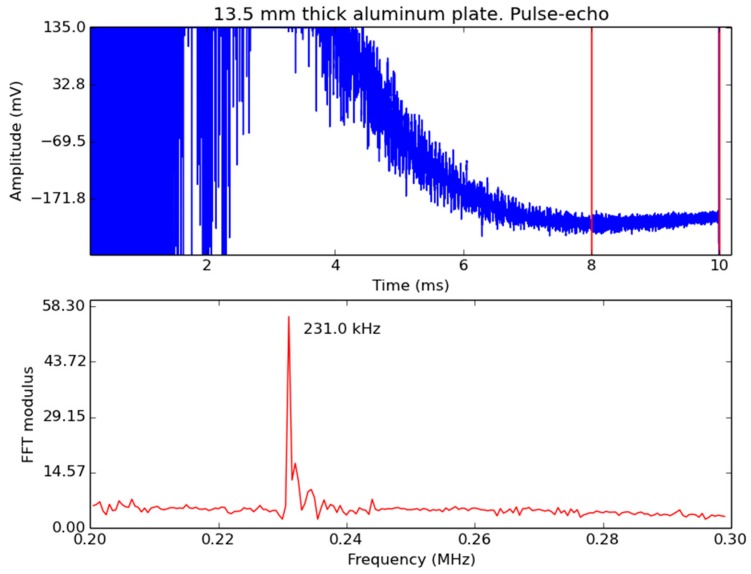
Pulse-echo; 13.5 mm-thick aluminum plate: signal in the time domain (**top**) and FFT modulus of the signal within the indicated window (**bottom**).

**Figure 11:**
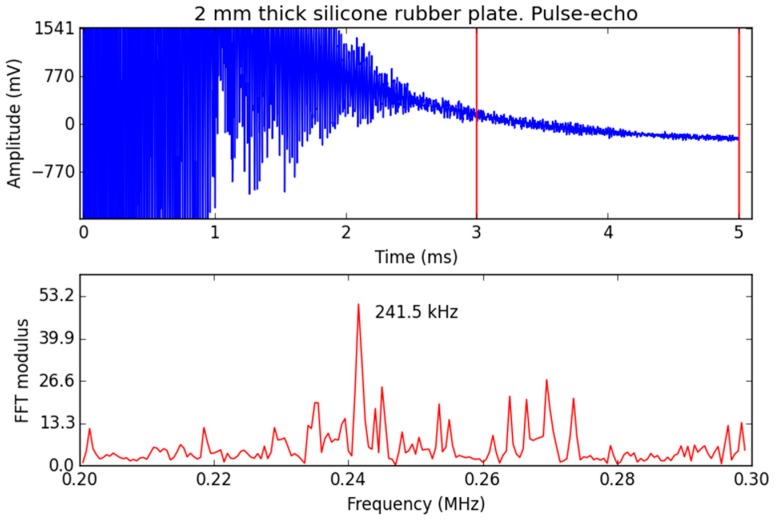
Pulse-echo; 2 mm-thick silicone rubber plate: signal in the time domain (**top**) and FFT modulus of the signal within the indicated window (**bottom**).

Resonant frequencies of the different samples obtained in PE mode (Table 4) agreed well with resonant frequencies measured in TT mode (Table 3). As expected from TT results, the largest signal amplitudes were obtained for the steel plates, while peak amplitudes in aluminum and silicone rubber plates were much smaller. Although peak amplitudes were similar in these two cases, there were some significant differences that were appreciated in Figure 10 and Figure 11. In the case of the silicone rubber plate (Figure 11), and because of the shorter *τ* (*τ* = 0.26 ms), the only way to measure the resonant frequency was to locate the temporal window for the FFT as close to *t_DZ_* (3 ms) as possible, otherwise the reverberations in the plate disappeared. Unfortunately, as we got closer to t*_DZ_*, the noise level increased, and some other frequencies appeared (resulting from the transducer band and the reverberations within the air cavity). On the contrary, for the aluminum plate (Figure 10) *τ* = 1.2 ms. We could further delay the temporal window (*t_o_* = 8 ms) and still have a relatively larger amplitude of the resonance peak. Only for the steel (1) pipe with a thickness 10.3 mm was no thickness resonance observed (Table 4). This was attributed to the larger roughness of the lathed wall in this section and to the fact that the expected resonant frequency was 276 kHz; at this frequency, the transducer sensitivity was slightly smaller (Figure 1).

#### 3.2.2. Pipes

**Figure 12:**
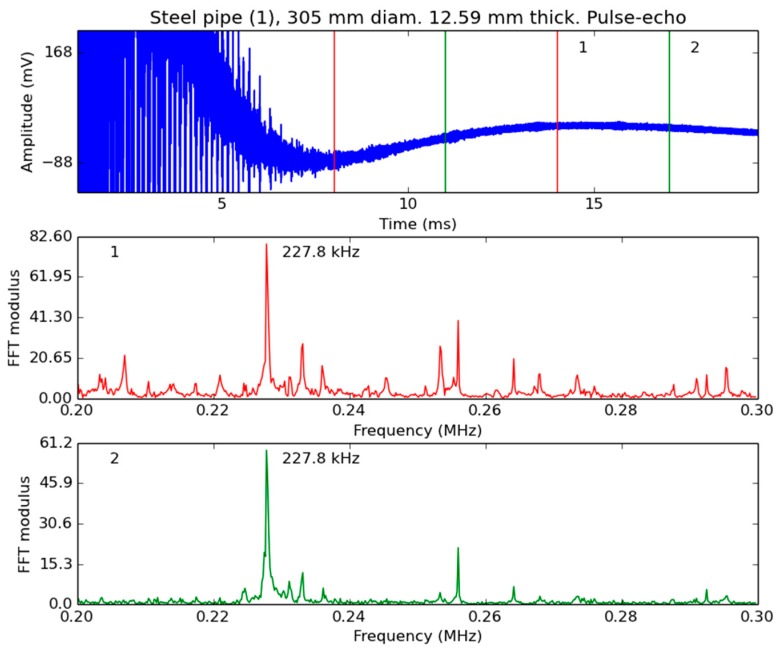
Pulse-echo; 12.59 mm-thick stainless steel pipe. Top: signal in the time domain. Center: FFT of the signal within window (1). Bottom: FFT of the signal within window (2).

For the steel pipes, and because of the geometrical reasons previously mentioned, the amplitude of the resonance peak was much smaller than in the case of plates. The larger amplitude corresponded to the steel (1) pipe in the section that was not lathed. This could be explained by the larger surface roughness present in the lathed sections and the negative influence of this roughness on the resonance condition. Figure 12 shows the measured response of the 305 mm diameter and 12.59 mm-thick steel (1) pipe. As in Figure 11, Figure 12 shows that selecting *t*_0_ close to *t_DZ_* resulted in an increase of the background noise in the FFT. However, and thanks to the larger value of *Z* and lower value of *α*, *τ* was larger. It was possible to use larger values of *t*_0_ with a moderate decrease in the amplitude of the resonance peak while eliminating most of the noise from other frequency components. In addition, Figure 12 revealed the presence of a much smaller peak that did not seem to be affected by the location of the FFT window, which appeared at 254 kHz. If we assume that this peak corresponds to the second-order thickness resonance of the shear wave, the velocity of this wave can be obtained from [24]:

(5) $$f_{res}^{n}\approx \;\frac{v}{2t}n$$

where *n* = 1, 2, … is the order of the resonance, which gives *v_t_* = 3200 m/s for *n* = 2, and it is consistent with known values for steel and with the previously measured longitudinal velocity.

#### 3.2.3. Influence of the Angle of Incidence

**Figure 13:**
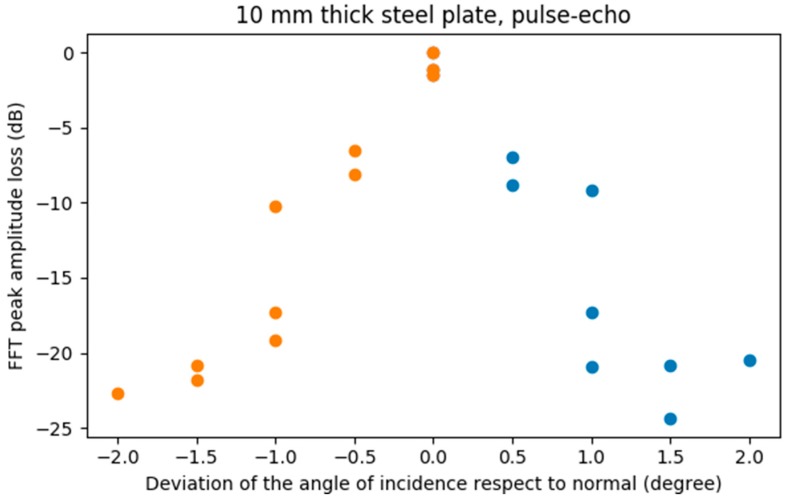
FFT peak loss in dB (compared with normal incidence peak amplitude) vs. deviation of the angle of incidence away from normal incidence for the 10 mm-thick stainless steel plate. The 14 ms window located at *t*_0_ = 6 ms.

Finally, we investigated the influence of possible misalignments between the transducer and plate on the amplitude of the resonance peak. To have an initial estimation of how significant this effect can be, we used the 10 mm-thick steel plate and deviated the angle of incidence from 0° (which corresponds to normal incidence) up to ±2° in steps of 0.5°. The variation in peak amplitude relative to the peak amplitude at normal incidence is shown in Figure 13. Differences at constant angles were due to the measurement noise.

**Figure 14:**
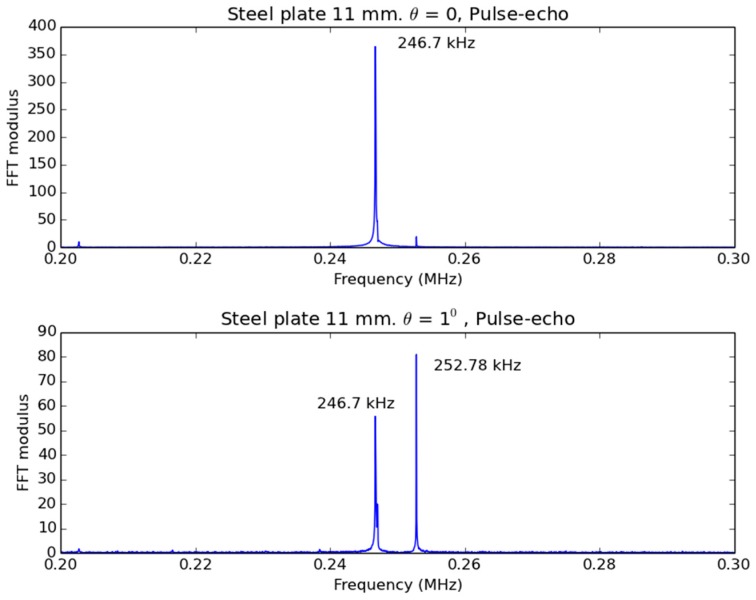
FFT modulus for the received signal in PE mode using a square temporal window: *t*_0_ = 20 ms, Δ*t* = 19 ms for the 11 mm-thick stainless steel plate, and two values of the deviation of the angle of incidence away from normal incidence: 0° (**top**) and 1° (**bottom**).

When incidence deviated from normal incidence, the amplitude of the resonance peak was expected to decrease. This was produced by the deviation of the ultrasonic beam from the transducer aperture as well as by the mode conversion and generation of shear waves in the plate surfaces. Appearance of shear waves was suggested to explain the additional peak observed in stainless steel pipes (Figure 12). This was better observed in this case thanks to the flat surfaces of the plate samples. Figure 14 shows the amplitude of the FFT in PE mode for the 11 mm-thick steel plate using a square temporal window, where *t*_0_ = 20 ms, Δ*t* = 19 ms, and for two different values of the deviation of the angle of incidence away from normal incidence (θ): 0° and 1°. The additional peak at 252.78 kHz for θ = 1° was due to the second-order resonance of the shear wave (vs. ≈ 2780 m/s).

Applying Equation (5), $f_{res}^{1}$ for longitudinal waves was 246.7 kHz, and $f_{res}^{2}$ for shear waves was 252.78 kHz. Then, the ratio of longitudinal to shear wave velocities $\left(r=v_{L}/v_{S}\right)$ was equal to 2×252.78 kHz/246.7 kHz. Poisson’s ratio (σ) can be calculated using Equation (6):

(6) $$\sigma =\frac{2r-1}{2\left(r-1\right)}$$

The obtained result for the stainless steel plate was σ = 0.32. If we applied the same procedure to the two resonant frequencies observed in the steel pipe (Figure 12), we obtained σ = 0.275. Reported data in the literature were σ = 0.3–0.31 for stainless steel [25,26] and σ = 0.27–0.30 for steel [26,27]. More accurate results could be obtained by replacing Equation (5) with the exact calculation of the location of the resonant frequencies for non-normal incidence.

### 3.3. Pulse-Echo C-Scan

**Figure 15:**
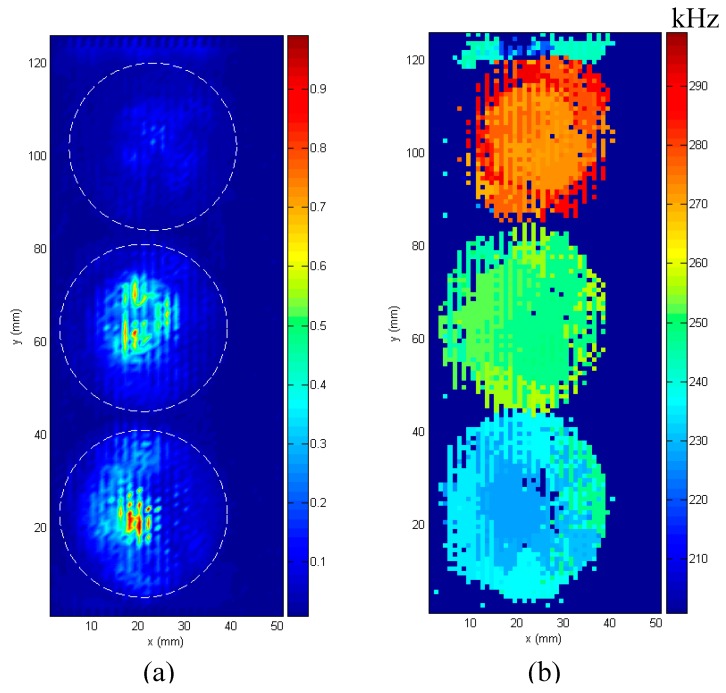
(**a**) C-Scan-normalized amplitude image of the three steel discs and (**b**) C-Scan image of the FFT center frequency. The colored bar in the second case represents the frequency in kHz.

Figure 15a shows the normalized amplitude C-Scan of the acquired data, and the approximate locations of the discs are overlaid in white. Maximum resonance amplitude was given at the center, where the behavior was more like that of an infinite plate when compared with the transducer diameter. When moving to the disc edges, nonsymmetry and border effects reduced the resonance amplitude. Amplitude differences between discs were explained by transducer response variations with frequency. Furthermore, Figure 15b shows an increase in resonant frequency near the disc edges when compared to the center region, which could be produced by the influence of the sample edge. Table 5 shows the average frequency values of the whole discs and in the central region only, where similar values to those in plates were obtained (Table 5).

## 4. Discussion and Conclusions

A resonant pulse-echo and air-coupled technique for high-impedance and low-attenuating materials as well as for specimens with regular geometry has been presented. The main output of this technique is the resonant frequency of the specimen that can be used to determine specimen dimensions (if the material or the ultrasound velocity is known) or to determine velocities (both longitudinal and shear waves) if the dimensions are known. This can be used for material characterization (to determine elastic moduli and Poisson’s ratio) or for NDT (to determine wall thickness and presence of pipe wall thinning or fouling). 

The technique was applied to plates at normal incidence to generate resonances of the longitudinal wave in the plate thickness in the frequency range 0.2–0.3 MHz, which allowed thicknesses to be measured from approximately 9 to 15 mm in aluminum, steel, and stainless steel, and 1.6 to 2.4 mm in rubber. Results were confirmed by comparing measured resonant frequency values with those obtained using a conventional through-transmission technique. The best results were obtained for stainless steel plates. As impedance of the material decreased, or the ultrasound attenuation coefficient increased, the performance of the technique decreased, which was exemplified by a decrease in the amplitude of the resonance peak and a decrease in the signal-to-noise ratio. Silicone rubber plates represented the limit of this technique’s applicability, where the resonance peak was barely above the noise level.

Influence of the angle of incidence was investigated. A decrease of 20 dB in resonance peak amplitude, for variations of the angle of incidence of ±2° away from the normal, was observed in stainless steel plates. This reduction was produced by deviation of the ultrasonic beam away from the transducer aperture and by the generation of shear waves. Oblique incidence was used to observe thickness resonances of both longitudinal waves (first-order thickness resonance) and shear waves (second-order thickness resonance) that could be used to determine the value of both longitudinal and shear wave velocities if thickness was known. If thickness was not known, then it was still possible to obtain Poisson’s ratio; values of 0.32 and 0.275 were obtained for stainless steel and steel, respectively.

The technique was also applied to cylindrical pipes with a diameter much larger than the transducer’s aperture. In this case, damping of the resonance was larger than in plates at normal incidence because of mode conversion and deviation of the beam away from the transducer’s aperture. In spite of these problems, it was possible to detect the pipe wall thickness resonance using this pulse-echo resonant technique in 305 and 324 mm diameter cylindrical steel pipes with wall thickness between 10 and 13 mm. In these cases, the shear wave resonance was always present because we did not have normal incidence over the whole beam aperture. 

The technique could be used to obtain a C-Scan-type image of a component thickness by color coding the resonant frequency of the signal spectrum. 

## Figures and Tables

**Table 1:** Circular plates used for the study: materials and thicknesses.

Material	Thickness (mm)	Diameter (mm)
Stainless steel	10.0 ± 0.05	36
	11.0 ± 0.05	36
	12.0 ± 0.05	36
Aluminum	13.5 ± 0.05	55
Silicone rubber	2.0 ± 0.02	40

**Table 2:** Pipes used for the study: material pipe diameters and wall thicknesses.

Material	Diameter/Wall Thickness (mm)
Steel (1)	305/10.30 ± 0.2
	305/11.16 ± 0.3
	305/11.86 ± 0.4
	305/12.59 ± 0.4
Steel (2)	324/13.10 ± 0.2

**Table 3:** Through-transmission resonant frequency.

Sample	Material	Thickness (mm)	Resonant Frequency (kHz)
Plate	Stainless Steel	10.0 ± 0.05	272.0
		11.0 ± 0.05	247.0
		12.0 ± 0.05	227.1
	Aluminum	12.5 ± 0.05	231.0
	Silicone rubber	2.0 ± 0.02	241.0
Steel Pipe	(1)	10.3 ± 0.2 ^1^	276
		11.16 ± 0.3 ^1^	257
		11.86 ± 0.4 ^1^	243
		12.59 ± 0.4	227.5
	(2)	13.1 ± 0.2	221.5

^1^ Lathed inner surface.

**Table 4:** Pulse-echo resonant frequency and peak amplitude.

Sample	Material	Thickness (mm)	Resonant Frequency ^2^ (kHz)	Peak Value of the Modulus of the FFT ^2^
Plate	Stainless Steel	10.0 ± 0.05	272.0	1197
		11.0 ± 0.05	247.0	1830
		12.0 ± 0.05	227.1	910
	Aluminum	12.5 ± 0.05	231.0	56.1
	Silicone rubber	2.0 ± 0.02	241.5	53.2
Pipe	Steel (1)	10.3 ± 0.2 ^1^	--	--
		11.16± 0.3 ^1^	257.1	17.0
		11.86 ± 0.4 ^1^	242.3	16.0
		12.59 ± 0.4	227.5	40.5
	Steel (2)	13.1 ± 0.2	221.7	14.0

**^1^** Lathed inner surface; **^2^** using a 2 ms rectangular window located after *t_DZ_*.

**Table 5:** Pulse-echo resonant frequencies at three discs in Figure 15.

Disc Thickness (mm)	Average Resonant Frequency (kHz)	Average Resonant Frequency at Center (r < 9 mm) (kHz)
10.0 ± 0.05	277.6 ± 6.9	272.9 ± 2.5
11.0 ± 0.05	249.9 ± 3.5	248.5 ± 2.4
12.0 ± 0.05	233.8 ± 6.1	229.2 ± 3.5
